# Dosimetric validation of a magnetic resonance image gated radiotherapy system using a motion phantom and radiochromic film

**DOI:** 10.1002/acm2.12088

**Published:** 2017-04-24

**Authors:** James M. Lamb, John S. Ginn, Dylan P. O'Connell, Nzhde Agazaryan, Minsong Cao, David H. Thomas, Yingli Yang, Mircea Lazea, Percy Lee, Daniel A. Low

**Affiliations:** ^1^ Department of Radiation Oncology David Geffen School of Medicine University of California Los Angeles CA USA; ^2^ Department of Radiation Oncology University of Colorado Denver Denver CO USA; ^3^ Computerized Imaging Reference Systems, Inc. Norfolk VA USA

**Keywords:** motion management, MRI, quality assurance, radiotherapy

## Abstract

**Purpose:**

Magnetic resonance image (MRI) guided radiotherapy enables gating directly on the target position. We present an evaluation of an MRI‐guided radiotherapy system's gating performance using an MRI‐compatible respiratory motion phantom and radiochromic film. Our evaluation is geared toward validation of our institution's clinical gating protocol which involves planning to a target volume formed by expanding 5 mm about the gross tumor volume (GTV) and gating based on a 3 mm window about the GTV.

**Methods:**

The motion phantom consisted of a target rod containing high‐contrast target inserts which moved in the superior‐inferior direction inside a body structure containing background contrast material. The target rod was equipped with a radiochromic film insert. Treatment plans were generated for a 3 cm diameter spherical planning target volume, and delivered to the phantom at rest and in motion with and without gating. Both sinusoidal trajectories and tumor trajectories measured during MRI‐guided treatments were used. Similarity of the gated dose distribution to the planned, motion‐frozen, distribution was quantified using the gamma technique.

**Results:**

Without gating, gamma pass rates using 4%/3 mm criteria were 22–59% depending on motion trajectory. Using our clinical standard of repeated breath holds and a gating window of 3 mm with 10% target allowed outside the gating boundary, the gamma pass rate was 97.8% with 3%/3 mm gamma criteria. Using a 3 mm window and 10% allowed excursion, all of the patient tumor motion trajectories at actual speed resulting in at least 95% gamma pass rate at 4%/3 mm.

**Conclusions:**

Our results suggest that the device can be used to compensate respiratory motion using a 3 mm gating margin and 10% allowed excursion results in conjunction with repeated breath holds. Full clinical validation requires a comprehensive evaluation of tracking performance in actual patient images, outside the scope of this study.

## 1 Introduction

Radiation therapy provides an effective way to combat numerous types of cancer, improving both a patient's quality and quantity of life. However, the effectiveness of a radiation treatment is limited by the accuracy and precision of the treatment's delivery. One of the greatest challenges limiting the accurate and precise delivery of a treatment plan is patient motion arising from respiratory, cardiac and gastrointestinal peristaltic processes. Numerous respiratory motion management techniques have been developed^1^, such as tumor tracking, 4D‐CT based internal target volumes, and respiratory gating. Extensive clinical experience exists for respiratory gating performed using external surrogates^2^, ^3^, ^4^ and implanted fiducial markers^5^, ^6^, ^7^. Surrogate‐based tracking suffers from the fact that the relationship between the surrogate and the tumor position may vary in time^8^, ^9^, ^10^. Implanted fiducials are associated with a small but non‐negligible risk of injury, cannot be easily implanted in all anatomic locations subject to respiratory motion, may change position in the body over time, and cannot easily capture target deformation^11^, ^12^, ^13^, ^14^. Magnetic resonance image (MRI) guided radiotherapy enables gating directly on target position for soft‐tissue targets in the lung and abdomen without implanted markers^15^. Currently, the only FDA‐approved MRI‐guided radiotherapy system is the ViewRay MRIdian (ViewRay, Inc., Cleveland, OH, USA). In this paper, we present a dosimetric evaluation of the MRIdian's respiratory gating performance using an MRI‐compatible motion phantom and radiochromic film. Our methodology is to perform static, gated moving, and ungated moving deliveries of the same plan and evaluate the gating performance in terms of its ability to “freeze” motion, i.e., quantitate the difference between each case and the static treatment plan using the gamma^16^ metric. This methodology has been frequently used to evaluate gating systems^17^, ^18^, ^19^, ^20^, ^21^, ^22^, ^23^. The purpose of this study was to establish a technical performance baseline of the respiratory gating which can inform clinical gating protocols as well as be used as a machine performance benchmark by other institutions.

The MRIdian can gate treatments based on planar images acquired during treatment. The gating is based on tumor tracking by deformable image registration. Real‐time images can be acquired in a single sagittal plane at 4 Hz or in three sagittal planes at 2 Hz. Because the additional 0.25 s of beam‐off latency required when using three‐plane imaging, our institutional policy is to not use three‐plane imaging, and it was not evaluated in this study. Our institution's clinical gating protocol uses the MRIdian's gating capabilities as follows. The gating target is contoured on the volumetric scan used for treatment planning. The gating target is usually the gross tumor volume (GTV), but could be a critical organ at risk or a surrogate structure. For best performance, the gating target contour should correspond to a strong contrast boundary in the image. For every treatment fraction, the target contour is transferred either rigidly or deformably to a volumetric pretreatment setup image. The target contour is optionally updated manually. Both the setup image and the simulation image are acquired under voluntary breath hold of 17 or 25 s duration depending on the imaging field of view. Subsequently, a two‐dimensional cine preview scan is acquired with a frame rate of four frames per second in a single sagittal plan centered on the tumor. The cine preview scan is acquired under free breathing conditions. Each frame of the preview scan is deformably registered to the volumetric setup image by the MRIdian software. Contours are propagated from the setup image to the best‐matching cine frame, called the key frame. During treatment, the now two‐dimensional gating target contour is propagated from the key frame to each cine image in real time. If the propagated target contour exceeds a preset boundary by a configurable percentage of its area, the treatment beam is held. The boundary corresponds either to a numerical expansion relative to the gating target's initial location, or to another contour from the planning volumetric image (e.g., the planning target volume).

Our clinical protocol uses repeated breath holds throughout the treatment duration. The MRIdian system tracks the target and enables beam only when the target is within a predefined boundary; repeated breath holds are used to maximize the duty cycle and minimize dosimetric error due to beam hold latency. A margin of 3 mm around the gross tumor volume (GTV) position in the volumetric setup scan is used as the gating boundary, and a 10% excursion of the tracked GTV contour outside the boundary is allowed. The 10% allowed excursion is used to make the gating procedure robust to random errors in contour tracking arising from image noise.

Repeated breath holds are used by preference if the patient is able to perform them at consistent states of inhalation, with coaching if needed. If the patient is not able to perform consistent repeated breath holds, free breathing is used at the discretion of the attending physician. If the gating efficiency is sufficiently low that the patient may not be able to tolerate the treatment time, the gating margin may be relaxed from 3 mm to 5 mm at the discretion of the attending physician.

## 2 Materials and methods

### 2.A Motion phantom

**Figure 1:**
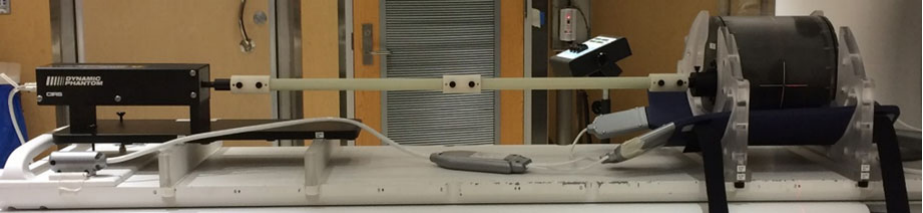
Side view of the CIRS MRI‐compatible motion phantom.

**Figure 2:**
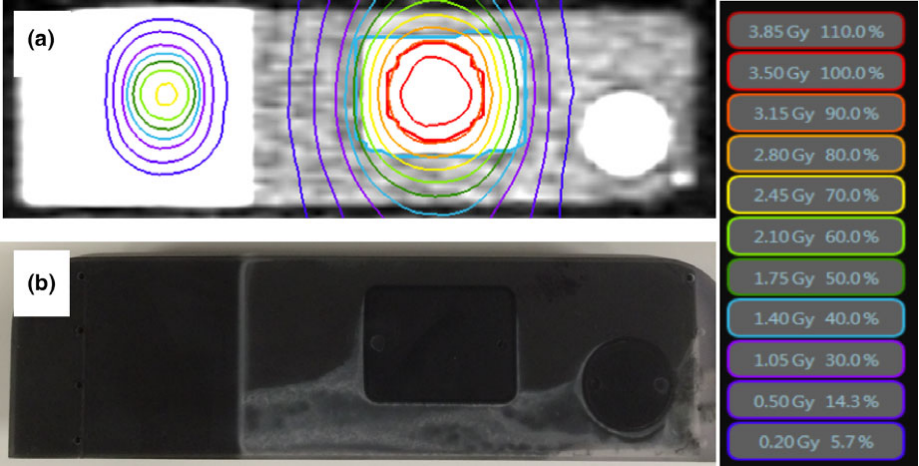
Target rod cross‐section MRI image with isodose distribution superimposed (a) and optical digital camera image (b). The reference beam appears on the left and the PTV on the right in this view. The PTV is a 3 cm diameter spherical target centered about the cuboid gating target.

Respiratory motion was simulated using a MRI‐compatible phantom that was capable of one‐dimensional motion in the superior‐inferior direction (Fig. 1). The phantom was developed by CIRS, Inc (Norfolk, VA, USA), with input from ViewRay and is commercially available from CIRS. The phantom consists of a stationary body structure filled with background contrast material and four small spheres of high‐contrast material. A 6.5 cm diameter target rod was made to move within the body structure by a stepper motor. The target rod included three regions of high‐contrast material (a small sphere, a small cuboid, and a large cylinder) within and adjacent to a background contrast region. In standard configuration, the target rod includes an ion chamber insert. A custom target rod with a film insert was developed by CIRS to our specification. A cross‐section of the target rod is shown in Fig. 2. The entire body structure, excluding a 1 cm acrylic supporting shell, is approximately water equivalent.

### 2.B Treatment planning

A treatment plan was produced using the ViewRay treatment planning system and delivered on the MRIdian system. The planning target volume (PTV) was a 3 cm diameter sphere. The plan contained 15 coplanar beams and the dose distribution was typical for a stereotactic body radiotherapy plan, scaled to a maximum dose 3.75 Gy per fraction to lie within the dynamic range of EBT3 film. In addition, a 2.1 cm × 1.05 cm reference beam delivering 2.4 Gy per fraction at the film surface was added at a 6.5 cm longitudinal isocenter shift relative to the PTV isocenter. The reference beam was used for film spatial registration and dose calibration as described below. Figure 2a shows the dose distribution at the surface of the film.

### 2.C Radiochromic film dosimetry

Gafchromic EBT3 radiochromic film (Ashland, Inc, Bridgewater, NJ, USA) was loaded in the phantom and carefully aligned to a raised fiducial reference line in the insert. To minimize the uncertainty in alignment due to cutting the film, the side of the film that was not cut was aligned to this reference line. FilmQA Pro 2015 (Ashland, Inc, Bridgewater, NJ, USA) with the triple‐channel algorithm^24^ was used for all film analysis. Calibration films from the same lot of film used for experiments were exposed at nine dose levels between 0 and 500 cGy using the MRIdian machine. Film was read using an Epson 10000 XL scanner (Epson America, Inc., Long Beach, CA, USA) at least 24 hr after irradiation. Delivered dose distributions were compared to the planned dose distributions using the gamma technique^16^, with 3 mm distance to agreement and 3%, 4%, and 5% global dose difference tolerance, and regions with dose less than 30% of the maximum dose were suppressed.

A 2.1 cm × 1.05 cm reference beam at a distance of 6.5 cm from the PTV isocenter was delivered with the phantom at rest after every motion trajectory. The reference beam was used to spatially register the film to the treatment plan in the software used for film dosimetry FilmQA. The reference beam was also used to extract a relative dose scaling factor to account for inter‐scan differences in film calibration. The spatial registration and the dose scaling factor were derived by iterative maximization of the gamma passing rate in an ROI including only the reference beam.

### 2.D Breathing waveforms

**Figure 3:**
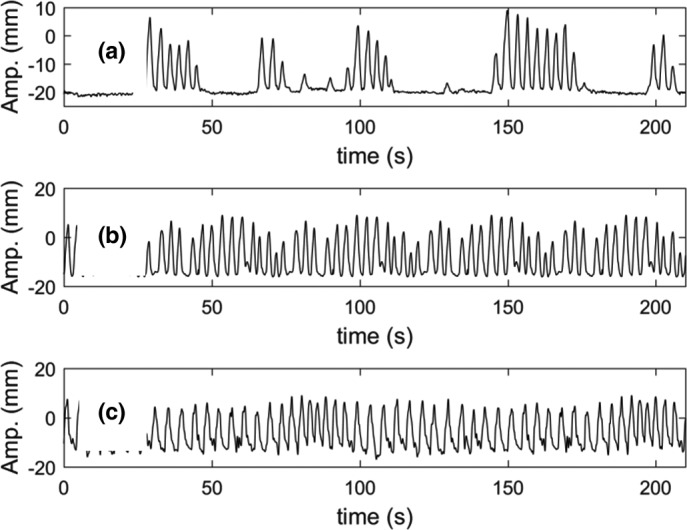
Patient tumor motion trajectories used in this study, including repeated breath hold (a) and free breathing (b) and (c).

An artificial breathing waveform at two frequencies and three actual tumor trajectories were used for phantom motion. The artificial breathing wave was a cos^6^ function, which was used to represent an idealized human breathing pattern which was asymmetric with more time spent at exhalation than inhalation. Trajectory amplitude was 2 cm peak‐to‐peak and both 10 and 15 breaths per minute frequencies were used. The actual tumor trajectories were derived from real‐time MRI imaging acquired at four frames per second during abdominal radiotherapy treatments on the MRIdian machine, and are shown in Fig. 3. One trajectory was acquired during repeated voluntary breath hold at exhalation, and the other two were acquired during free breathing. To test the robustness of gating to rapid breathing, sped‐up versions of each trajectory were also tested. Trajectory properties are listed in Table 1. Trajectories were looped throughout the duration of treatment delivery.

**Table 1:** Characteristics of breathing waves

N	Description	Amplitude (5th–95th)	Duration	Mean period
1	Repeated Breath hold	19 mm	210 s	NA
2	Free breathing 1	21 mm	45 s	3.2 s/breath
3	Free breathing 2	19 mm	111 s	4.0 s/breath
4^a^	Fast Repeated breath hold	19 mm	105 s	NA
5^a^	Fast free breathing 1	21 mm	23 s	1.6 s/breath
6^a^	Fast free breathing 2	19 mm	28 s	1.0 s/breath

a Trajectories 4, 5, and 6 are copies of trajectories 1, 2, and 3 sped up by factors of 2, 2, and 4, respectively.

## 3 Results

For the static delivery, 96.5% of points passed the gamma criteria at 3%/3 mm tolerance. Dosimetric accuracy relative to the static treatment plan under different motion trajectories and with different gating parameters is shown in Table 2. Using 3 mm gating window and 10% allowed excursion, the breath hold trajectory at actual speed had 97.8% gamma pass rate at 3%/3 mm criteria, and all of the patient tumor motion trajectories at actual speed had at least 95% gamma pass rate at 4%/3 mm. Dosimetric accuracy for the artificially sped‐up trajectories is shown in Table 3. Accuracy was noticeably less for the artificially sped‐up trajectories and for the sinusoidal trajectories. Considering all trajectories, increased accuracy is observed for 3 mm versus 5 mm gating margin (*P* < 0.004) and for 5% allowed excursion versus 10% allowed excursion (*P* = 0.005). Statistical testing was performed with three‐way ANOVA using margin size, allowed excursion, and breathing trajectory as grouping variables.

**Table 2:** The individual exposure results of gating experiments with patient tumor motion trajectories and sinusoidal trajectories

Trajectory	Gating parameters	Duty cycle	Gamma 3%, 3 mm	Gamma 4%, 3 mm	Gamma 5%, 3 mm
Breath hold	None	100%	49.9%	59.4%	66.9%
Breath hold	3 mm, 5%	78.3%	95.2%	98.8%	100%
Breath hold	3 mm, 10%	81.5%	97.8%	99.2%	100%
Breath hold	5 mm, 10%	80.6%	89.5%	96.5%	99.8%
Free breathing 1	None	100%	25.0%	27.7%	30.6%
Free breathing 1	3 mm, 5%	56.7%	91.7%	98.2%	100%
Free breathing 1	3 mm, 10%	61.9%	91.6%	98.2%	100%
Free breathing 1	5 mm, 10%	66.0%	81.0%	90.5%	93.8%
Free breathing 2	None	100%	32.9%	37.2%	41.9%
Free breathing 2	3 mm, 5%	56.9%	98.6%	100%	100%
Free breathing 2	3 mm, 10%	74.8%	89.5%	97.1%	99.8%
Free breathing 2	5 mm, 10%	71.5%	88.7%	95.8%	99.6%
Cos^6^ 10 bpm	None	100%	24.1%	26.3%	28.9%
Cos^6^ 10 bpm	3 mm, 5%	61%	87.13	95.45%	99.21%
Cos^6^ 10 bpm	3 mm, 10%	75%	76.6%	86.3%	94.15%
Cos^6^ 10 bpm	5 mm, 10%	72.9%	69.7%	79.19%	84.68%
Cos^6^ 15 bpm	None	100%	26.95	30.86	34.2%
Cos^6^ 15 bpm	3 mm, 5%	70%	64.85%	74.62%	84.96%
Cos^6^ 15 bmp	3 mm, 10%	68.8%	53.15%	60.99%	68.8%
Cos^6^ 15 bpm	5 mm, 10%	75.8%	42.04%	94.9%	57.96%

**Table 3:** The individual exposure results of gating experiments with artificially sped up measured tumor trajectories

Trajectory	Gating parameters	Duty cycle	Gamma 3%, 3 mm	Gamma 4%, 3 mm	Gamma 5%, 3 mm
Fast breath hold	None	100%	35.80%	41.45%	47.00%
Fast breath hold	3 mm, 5%	79%	94.4%	98.6%	99.8%
Fast breath hold	3 mm, 10%	87%	91.90%	97.60%	99.60%
Fast breath hold	5 mm, 10%	81%	88.30%	94.60%	98.60%
Fast free breathing 1	None	100%	19.40%	22.20%	24.20%
Fast free breathing 1	3 mm, 5%	54%	65.50%	75.95%	83.95%
Fast free breathing 1	3 mm, 10%	61%	49.50%	56.10%	63.50%
Fast free breathing 1	5 mm, 10%	70%	43.70%	50.50%	56.50%
Fast free breathing 2	None	100%	22.60%	26.40%	29.30%
Fast free breathing 2	3 mm, 5%	55.6%	88.85%	97.30%	99.20%
Fast free breathing 2	3 mm, 10%	60%	82.15%	91.50%	95.90%
Fast free breathing 2	5 mm, 10%	63%	71.80%	82.07%	88.03%

**Figure 4:**
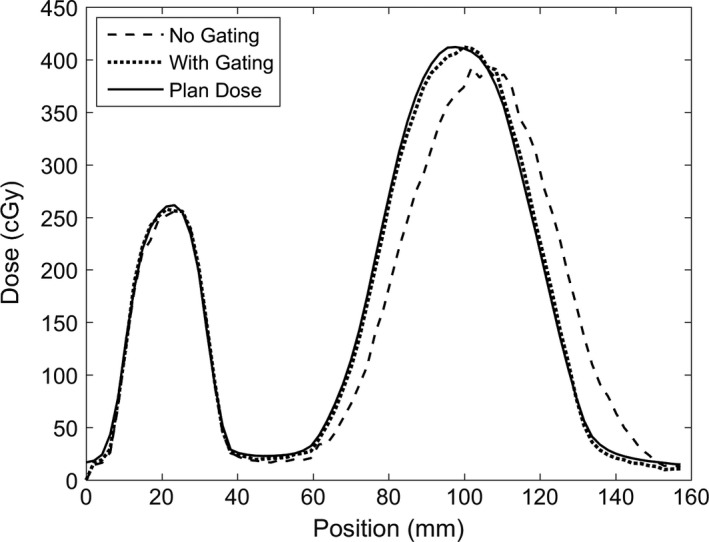
Measured gated and non‐gated, and static plan dose profiles on a line drawn through the center of the target volume. The small distribution on the left is the reference beam, and the larger distribution on the right is the PTV dose profile. The dose distribution broadens and shifts in the direction of motion when no gating is performed. The beam profile full widths at half maximum are 45 mm, 45 mm and 46 mm, respectively, for the planned, gated, and ungated dose profiles, respectively. The beam profile centers are 98 mm, 99 mm, and 104 mm, respectively for the three profiles.

The dose profile tool in FilmQA Pro was used to better understand how the gating affects the dose distribution in the inferior‐superior direction. Figure 4 shows the measured gated and nongated dose profiles as well as the planned dose profile in the direction of motion and through the center of the reference beam and the PTV. The motion profile used for this figure was a cos^6^ function with a period of 6 s and 2 cm amplitude. The gated exposure used a 3 mm gating margin with a 10% allowed excursion. Contrary to our initial expectation, a shift of the profile in the direction of motion appears more pronounced than the broadening of the dose profile. The recovery of the static distribution by gating using a 3 mm margin is well visualized.

## 4 Discussion

The gamma pass rate for our preferred clinical gating protocol (repeated breath holds, 3 mm gating margin, 10% excursion tolerance) was as good as the gamma pass rate of a static delivery using 3%/3 mm criteria, indicating that under such a protocol, phantom motion is completely compensated. For both free‐breathing measured tumor trajectories, 3 mm gating margin and 10% excursion tolerance resulted in pass rates above 95% for 4%/3 mm criteria. Our standard MRI‐gated protocol uses a 5 mm PTV margin, so a residual 2 mm of margin is available to account for motion‐unrelated uncertainty. Increasing the gating margin to 5 mm resulted in a statistically significantly reduction in accuracy, however, accuracy was still adequate in most cases, compared to an overall target dose uncertainty goal of 5%.

In the case of free breathing tumor trajectories that were artificially sped up to 1.0 and 1.6 s per breath, as well as the cos^6^ trajectories, dosimetric accuracy was reduced. Lowering the allowed excursion from 10 to 5% recovered the accuracy somewhat. It is important to note that this does not necessarily mean that a tumor moving in such a trajectory would be under‐dosed. Determining adequacy of coverage requires a comprehensive assessment of a complex set of uncertainties as well as consideration of the PTV margin, which is beyond the scope of this paper. Quantitative assessment of the dose accumulated to the CTV given a PTV margin is hampered by the fact that PTV margins are designed to compensate for multiple uncertainties in planning, setup and delivery, not just motion, as well as the lack of three‐dimensional information provided by a film measurement. Our result support the use of a PTV margin for patients treated with breath hold gating that is similar to those treated to static targets not subject to respiratory motion. Conversely, our results indicate that care should be taken in the case of very rapid breathers. A breathing rate of one breath per second would certainly be unusual, but not inconceivable. A limitation of our results is that the features of the phantom are idealized, and the contrast higher than that is observed in many patients, both of which may affect the accuracy of the tracking algorithm. A 30% threshold was used in the gamma pass rate computation because it is the clinical standard at our institution. To test the sensitivity of the results to the threshold value, the comparison was repeated with a 10% threshold for the four irradiations using the breath hold trajectory, yielding a gamma pass rate lower by 0.1–2.7% (mean:1.1%).

Inaccuracies in gating result from differences in gating surrogate versus true tumor position^8^, ^25^ and latency of beam on and beam off relative to gating signal. Real‐time MRI guidance allows gating on the actual tumor, effectively removing the first of these sources of uncertainty. The gating latency specification of the MRIdian system is a maximum beam‐off latency of 0.5 s including the effects of 4 frames/s imaging frequency, processing time, and source shuttle motion. Beam‐off latency on our system was measured to be 0.436 s (average of 33 measurements per head). Predictive filtering is not used by the MRIdian system. Other gating systems’ beam‐off latencies have been reported in the range of 0.044–0.529 s^26^, ^27^, ^28^, ^29^. For systems using predictive filtering, latency depends on breathing^26^. The dosimetric error resulting from gating latency can be controlled by minimizing the number of transitions between gated and un‐gated states, as in repeated breath hold gating. Our present study is limited in the number of tumor trajectories studied, and further work is needed to draw statistically significant conclusions regarding the sensitivity of dosimetric accuracy to the amplitude, rate, and waveform of patient breathing. The observations of decreased accuracy when using a 5 mm versus a 3 mm gating window, and increased accuracy when using a 5% versus a 10% allowed excursion were both statistically significant.

## 5 Conclusion

This study demonstrates a baseline performance of MRI‐guided gating with the ViewRay MRIdian and suggests that the device can be used to compensate respiratory motion using a 3 mm gating margin and 10% allowed excursion results in conjunction with repeated breath holds. The results of this study could be used by other institutions to inform clinical gating protocols and to benchmark machine performance. Clinical gating performance will depend on the accuracy of tracking in patients, which may not be as high as with the motion phantom used for this study. A detailed investigation of tracking performance in actual patients is warranted, but outside of the scope of this study. A characterization of accuracy under a comprehensive set of breathing waveforms would also be beneficial, because dosimetric accuracy under MRI gating depends on the amplitude and frequency of breathing‐induced motion.

## Conflict of Interest

Drs. Lamb, Cao, Yang and Lee report personal fees from ViewRay, Inc. Mr. Lazea is employed by CIRS, Inc.
